# Closing the bandgap for III-V nitrides toward mid-infrared and THz applications

**DOI:** 10.1038/s41598-017-11093-4

**Published:** 2017-09-06

**Authors:** Pengfei Lu, Dan Liang, Yingjie Chen, Chunfang Zhang, Ruge Quhe, Shumin Wang

**Affiliations:** 1 State Key Laboratory of Information Photonics and Optical Communications, Beijing University of Posts and Telecommunications, Beijing, 100876 China; 2 School of Information and Communication Engineering, Beijing University of Posts and Telecommunications, Beijing, 100876 China; 3 Beijing Computational Science Research Center, Beijing, 100193 China; 4 State Key Laboratory of Functional Materials for Informatics, Shanghai Institute of Microsystem and Information Technology, Chinese Academy of Sciences, Shanghai, 200050 China; 5 Photonics Laboratory, Department of Microtechnology and Nanoscience, Chalmers University of Technology, 41296 Gothenburg, Sweden

## Abstract

A theoretical study of InNBi alloy by using density functional theory is presented. The results show non-linear dependence of the lattice parameters and bulk modulus on Bi composition. The formation energy and thermodynamic stability analysis indicate that the InNBi alloy possesses a stable phase over a wide range of intermediate compositions at a normal growth temperature. The bandgap of InNBi alloy in Wurtzite (WZ) phase closes for Bi composition higher than 1.5625% while that in zinc-blende (ZB) phase decreases significantly at around 356 meV/%Bi. The Bi centered ZB InNBi alloy presents a change from a direct bandgap to an indirect bandgap up to 1.5625% Bi and then an oscillates between indirect bandgap and semi-metallic for 1.5625% to 25% Bi and finally to metallic for higher Bi compositions. For the same Bi composition, its presence in cluster or uniform distribution has a salient effect on band structures and can convert between direct and indirect bandgap or open the bandgap from the metallic gap. These interesting electronic properties enable III-nitride closing the bandgap and make this material a good candidate for future photonic device applications in the mid-infrared to THz energy regime.

## Introduction

Group III-V semiconductor compounds, as one of the most significant semiconductor materials^1, 2^, present bandgaps in a wide spectral range specially valued in optoelectronic systems and long wavelength photonic devices^3–8^. For example, III-nitride semiconductor compounds therein have been established successfully by Nakamura and co-workers^9, 10^ as a strategic material system for fabrication of blue light emitting diodes (LEDs). And notably, the narrow bandgap material indium nitride (InN) can be tuned by doping other III or V element into the host to achieve an extent potential application prospect in optoelectronic and high power/temperature electronic devices including LEDs, laser diodes (LDs), solar blind photodetectors and heterostructure field effect transistors^11^. There was a report that by changing the Ga composition continuously in InGaN alloy, the bandgap was tuned from 0.8 eV (InN) to 3.5 eV (GaN)—a range that spans the whole visible spectrum^12^ and is specially used for construction of green-blue violet LEDs, blue-violet LDs, solar cells and detectors operating in the short-wavelength range^13^. Similar applications are found for InAlN with its direct gap covering the range from 0.7 eV (InN) to 6.1 eV (AlN), which makes the InAlN alloys good candidates for optical applications over a wide spectrum from deep ultraviolet (UV) to near-infrared (IR) region^13^. The abovementioned dopants are almost cationic, and few researches are reported about the anionic dopant of group-V element by its substitution at the N site which may result in the potential use in mid-IR or THz applications.

The largest group-V element of bismuth (Bi) reveals attractive effects on physical properties of III-V-Bi materials. Even a small amount of Bi added will generate a huge decrease in the bandgap and an increase in the spin-orbit splitting energy^14, 15^, as shown by alloying Bi in GaAs and InAs^15, 16^. Polak *et al*.^17^ employed density functional theory (DFT) and clearly demonstrated that both the conduction bands (CBs) and valence bands (VBs) of Ga-V and In-V compounds change with Bi incorporation up to 3.7%. Other investigations of ternary and quaternary III-V-Bi materials such as InAsBi, InSbBi, and InAsSbBi synthesized by molecular beam epitaxy and vapor phase epitaxy^18–20^ indicated that the bandgap decreases with the addition of Bi at a rate of 55 meV/%Bi for InAsBi and 46 meV/%Bi for InAsSbBi^20^. In these dilute-bismide alloys, the reduction of bandgap could be explained by hybridization of the occupied Bi *p*-orbitals with VB. In transport measurement, the VB hybridization mechanism was proposed^21^. It shows that there’s a reduction in hole mobility by an order of magnitude, while the reduction of electron mobility is very limited. Whereas, at a high Bi composition, the compositional disorder plays a major role.

In spite of that there are lots of theoretical and experimental works on properties of InN and its compounds^10–13^, to our knowledge, we haven’t found any study on nearly perfect InN-related material that can be applied to mid- and far-IR region. Since InN has a small bandgap about 0.7–0.9 eV and InBi exhibits metallic nature with a negative indirect bandgap, incorporation of a low Bi composition in InN is expected to achieve mid to far-IR emission. Considering the larger ionic radii of Bi atom than that of N atom and the largest difference in electronegativity among all the group-V elements, the substitution of Bi over N may lead to an enhancement in lattice parameters of InNBi. Besides, the impurity states introduced by Bi may have strong modification on the band structure of InN. So the heavily affected InNBi alloy could exhibit unusual behavior for optoelectronic properties and provide building blocks in the III-N family for widening applications toward mid-IR, far-IR and THz regime, thus closing the bandgap and enabling III-nitrides covering the whole spectrum from deep UV to THz.

In this paper, we perform DFT calculations for the structural, thermal stability and electronic properties for InNBi alloy by incorporating Bi atoms into wurtzite (WZ) and zinc-blende (ZB) InN, and discuss these properties of InN_1−x_Bi_x_ with different *x* compositions. The paper is organized as follows. In Sec. 2, we describe details of computational methods and structural models. The results and discussions are provided in Sec. 3. Finally, a brief summary is summarized in Sec. 4.

## Methods

All the theoretical calculations are carried out by using DFT^22^ of the projector augmented wave method (PAW)^23, 24^ as implemented in the Vienna *ab initio* simulation package (VASP)^25^. In the calculations of structural properties, the exchange-correlation interaction is treated with the generalized gradient approximation (GGA) of the Perdew-Burke-Ernzerhof (PBE)^26^. In order to overcome the underestimation of GGA potential on the bandgap of the electronic properties, we have used the modified Becke-Johnson exchange potential in combination with local density approximation correlation (MBJLDA)^27^, because of its accuracy in describing the electronic structures of semiconductors and insulators. The spin-orbit coupling (SOC) has a significant effect on Bi-containing compounds and therefore is included in the electronic calculations. In all the computations, the cutoff energy is chosen as 350 eV for the plane wave expansion of the wave-functions. The structural optimization is allowed to relax until the maximum force on each atom becomes less than 0.01 eV/Å and maximum energy change between two steps is smaller than 10^−5^ eV. The Monkhorst-Pack grid is gamma-centered and several high symmetry k-points are used in the band structure calculations. A Monkhorst-Pack of 4 × 4 × 4 k-point mesh is used in the first Brillouin zone.

**Figure 1:**
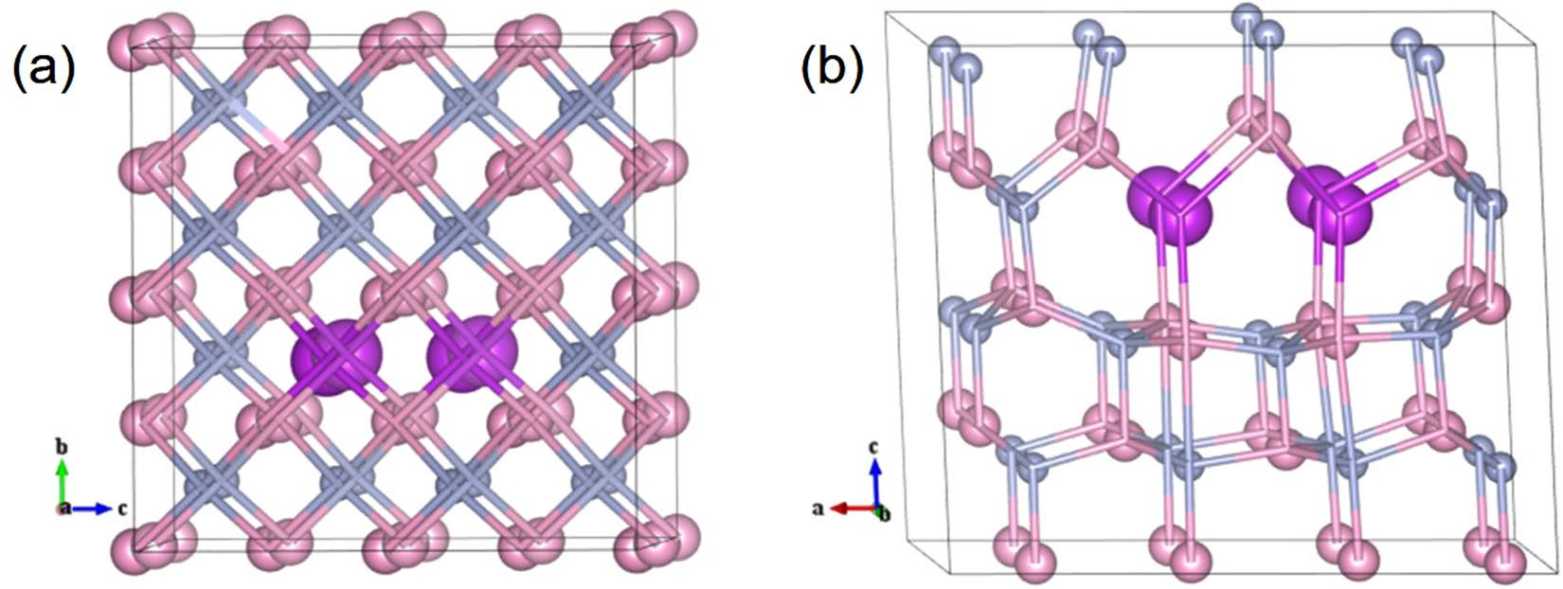
Schematic arrangement of atoms for clustered InN_0.875_Bi_0.125_ alloy in (**a**) ZB and (**b**) WZ phases, respectively.

The supercells containing 128 and 64 atoms for ZB and WZ phases are used to generate different Bi compositions for InNBi alloy. The Bi composition of 1.5625% is realized by substituting one N atom with a Bi atom at a nearly centered site in a 128-atom supercell, and other compositions of 3.125%, 6.25%, 12.5%, 25%, 50% and 75% are realized by substituting 1, 2, 4, 8, 16 and 24 N atoms by the same number of Bi atoms in a 64-atom supercell. For a given composition, an extreme atomic arrangement by clustering Bi atoms on anion sites is considered (clustered alloy). There is no unique procedure to obtain the most clustered structures, take InN_0.875_Bi_0.125_ as an example, the clustered structures for ZB and WZ are shown in Fig. 1, respectively, where every fourth anion hexagonal layer consists entirely of Bi atoms^28^.

## Results

### Geometry-based properties of InNBi

The optimized InNBi alloy structures of different Bi compositions are obtained by minimization of the total energy for the corresponding supercell. The equilibrium lattice constant and bulk modulus of different structures determined by fitting the energy versus volume into the Birch-Murnaghan equation of states^29^ are presented in Table 1 for ZB and WZ phases, together with other theoretical and experimental data. The calculated lattice constant of pristine InN in ZB phase is 5.100 Å, those of pristine InN in WZ phase are 3.570 Å for *a* and 5.741 Å for *c*, and that of pristine InBi in ZB phase is 6.849 Å, all of which are in good agreement with previous results. For InBi in WZ phase, we obtain the lattice constant *a* is 4.899 Å and *c* is 7.785 Å. From Table 1, we observe that the lattice constant of the InNBi alloy rises and the bulk modulus declines with increasing Bi composition, in accordance with the relationship between the unit cell volume and its bulk modulus. This behavior may come from the fact that the increase of Bi composition causes less hardness in these alloys, which may provide valuable information for experiments. In Supplementary Fig. S1, we display the lattice constant *a* and *c* as a function of Bi composition together with Vegard’s law^30^, where a clear deviation from this law is shown.

**Table 1:** Lattice constant and bulk modulus of InN_1−x_Bi_x_ in this work together with values from other calculations and experiments for comparison.

*x*	*ZB*		*WZ*		
	*a (Å)*	*B (GPa)*	*a (Å)*	*c (Å)*	*B (GPa)*
0	5.100, 5.109^a^, 5.040^b^, 4.98^c^ (exp)	97.741, 116.96^a^, 137^d^ (exp)	3.570, 3.501^e^, 3.614^a^, 3.544^f^ (exp)	5.741, 5.669^e^, 5.884^a^, 5.718^c^ (exp)	82.041, 116.1^a^ 125^d^ (exp)
0.015625	5.120	107.308	3.587	5.771	122.824
0.03125	5.145	87.048	3.602	5.800	81.658
0.0625	5.172	56.304	3.614	5.858	47.933
0.125	5.221	52.349	3.710	6.016	37.987
0.25	5.394	51.428	3.716	6.260	32.028
0.50	5.771	39.685	3.694	7.272	40.499
0.75	5.912	20.484	4.483	7.677	31.555
1.00	6.849, 6.838^g^	27.556, 33.87^g^	4.899	7.785	65.308

^a^Ref. 31, ^b^Ref. 11, ^c^Ref. 32, ^d^Ref. 33, ^e^Ref. 34,^f^ Ref. 35, ^g^Ref. 30.

### Chemical and thermo-stabilities

#### Binding energy and chemical stability

The formation energy of InN and InBi can be given by

1 $${\rm{\Delta }}{E}_{f}=E(InN)-E(In)-E(N)$$

2 $${\rm{\Delta }}{E}_{f}=E(InBi)-E(In)-E(Bi)$$

The formation energy of Bi doping into InN alloy can be written as,

3 $${\rm{\Delta }}{E}_{f}(In{N}_{1-x}B{i}_{x})=E(In{N}_{1-x}B{i}_{x})+xE(N)+x{\mu }_{N}-E(InN)-xE(Bi)-x{\mu }_{Bi}$$

where ${\rm{\Delta }}{E}_{f}(A)$ represents the formation energy of system A, $E({\rm{B}})$ is the energy of alloy B, ${\mu }_{N}$ and ${\mu }_{Bi}$ are the chemical potential of N and Bi, respectively.

For binary InBi alloy, there are two different ways to define the formation energy. One way is from In and Bi crystal, as shown by Eq. (2). The other way is by replacing N atoms in InN alloy which can be depicted by Eq. (3). The *µ*_*N*_−*µ*_*Bi*_ should be larger than zero for favorable Bi doping into InN and the upper limit for *µ*_*N*_−*µ*_*Bi*_ is obtained by assumption that the abovementioned both ways give the same formation energy for the InBi supercell.

**Figure 2:**
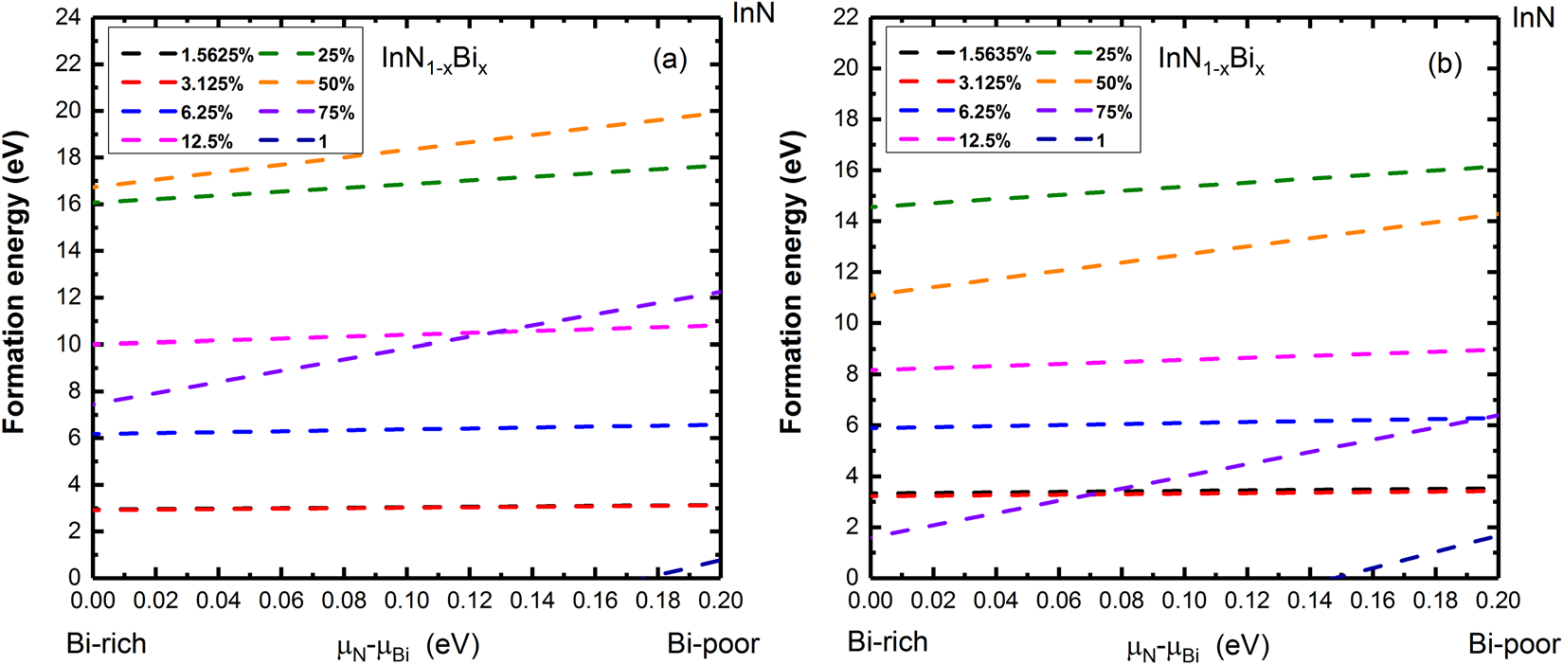
Formation energy of (**a**) ZB and (**b**) WZ-InN_1−x_Bi_x_ as a function of chemical potential difference of N and Bi.

For ZB phase, the formation energy of pristine InN and InBi is 0.183 and 0.017 eV, respectively, using GGA calculations. The stability of InNBi alloy in ZB phase can be described by the formation energy of InNBi alloy as a function of the chemical potential difference *µ*_*N*_−*µ*_*Bi*_ which has a lower limit of zero for the Bi rich condition and the upper limit of 0.180 eV for the Bi poor condition. As seen in Fig. 2(a), the formation energy of InN_1−x_Bi_x_ is almost independent of the chemical potential difference when the Bi incorporation level is lower than 25% and the most stable composition is at 50% which has the biggest formation energy than all other concentrations in the full *µ*_*N*_−*µ*_*Bi*_ range.

A similar analysis is performed for WZ phase, and the upper limit of *µ*_*N*_−*µ*_*Bi*_ is 0.184 eV for the Bi poor condition. As seen in Fig. 2(b), the formation energy at 25% is larger than all other concentrations in the full *µ*_*N*_−*µ*_*Bi*_ range, indicating the most stable composition is different from 50% in the ZB phase. The formation energy of InN_1−x_Bi_x_ is almost independent on the chemical potential difference.

#### Thermo-stability by temperature-composition diagram

In order to study phase stability of InNBi alloy, we investigate the mixing Gibbs free energy (Δ*G*_*m*_) and mixing enthalpy (Δ*H*_*m*_) of InN_1−x_Bi_x_ by using the calculations of GGA potential to obtain the *T-x* phase diagram^36, 37^ for differentiating stable, metastable and unstable mixing regions. The mixing Gibbs free energy for InN_1−x_Bi_x_ alloy is expressed as

4 $${\rm{\Delta }}{G}_{m}={\rm{\Delta }}{H}_{m}-T{\rm{\Delta }}{S}_{m}$$

with

5 $${\rm{\Delta }}{H}_{m}={\rm{\Omega }}x(1-x)$$

and

6 $${\rm{\Delta }}{S}_{m}=-R[x\,In\,x+(1-x)In(1-x)]$$

where $\Delta {S}_{m}$ is the mixing entropy, Ω is the interaction parameter, *x* is the Bi concentration, *R* is the gas constant, and *T* is the absolute temperature. The mixing enthalpy of InNBi can be obtained from the calculated total energies as

7 $${\rm{\Delta }}{H}_{m}={E}_{In{N}_{1-x}B{i}_{x}}-x{E}_{InBi}-(1-x){E}_{InN}$$

where ${E}_{In{N}_{1-x}B{i}_{x}}$, *E*_*InBi*_ and *E*_*InN*_ are the total energies for InNBi, InBi and InN, respectively.

By rewriting Eq. (5) as

8 $${\rm{\Omega }}={\rm{\Delta }}{H}_{m}/x(1-x)$$

for each *x*, we can calculate a value of Ω from the value of Δ*H*_*m*_. We summarize the calculated mixing enthalpy and interaction parameter for ZB and WZ phases in Table 2.

**Table 2:** The calculated mixing enthalpy (Δ*H*_*m*_ in Kcal/mole) and interaction parameter (Ω in Kcal/mole) of InN_1−x_Bi_x_ in ZB and WZ phases.

*x*	ZB		WZ	
	Δ*H*_*m*_	*Ω*	Δ*H*_*m*_	*Ω*
0.015625	0.135	8.836	0.150	9.786
0.03125	0.134	4.429	0.146	4.822
0.0625	0.283	4.828	0.268	4.578
0.125	0.464	4.246	0.380	3.373
0.25	0.757	4.039	0.682	3.639
0.50	0.847	3.389	0.583	2.333
0.75	0.506	2.699	0.229	1.189

**Figure 3:**
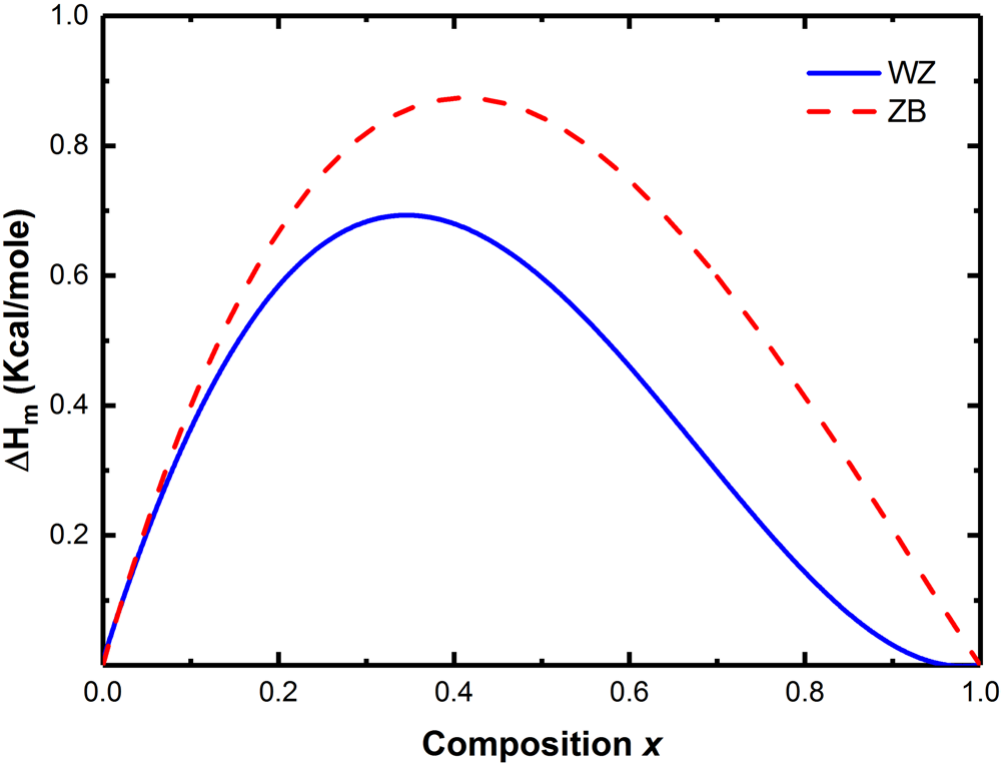
Mixing enthalpy Δ*H*_*m*_ as a function of composition *x* in InN_1−x_Bi_x_ with red dashed and blue solid lines for ZB and WZ phases, respectively.

From Table 2, we find that *Ω* has a significant composition dependence and decreases with increasing Bi composition in InNBi alloy for both phases. We also get a larger value of Ω (*x* = 0) than that of *Ω* (*x* = 1), which agrees with the fact that incorporating a small atom *A* into a host crystal of large *B* atoms (i.e., *x* → 1) requires less energy than incorporating a large atom *B* into a host crystal of small A atoms (i.e., *x* → 0) in the A_1−x_B_x_ system^38^. Table 2 also shows larger Δ*H*_*m*_ for ZB phase than those for WZ phase which is also drawn by the red and blue solid curves in Fig. 3. This figure shows the rapid increment of Δ*H*_*m*_ up to a maximum value at about *x* ~ 0.4 and then a monotonic decrease for both phases. Besides, the mixing enthalpies are all positive for both phases, which means that the system has a strong tendency to segregate in its constituents at low temperature. At high temperature, it is expected that the disordered configurations become favored due to the important increase of the entropic term. Our aim here is to determine the behavior of the alloy between such limits.

**Figure 4:**
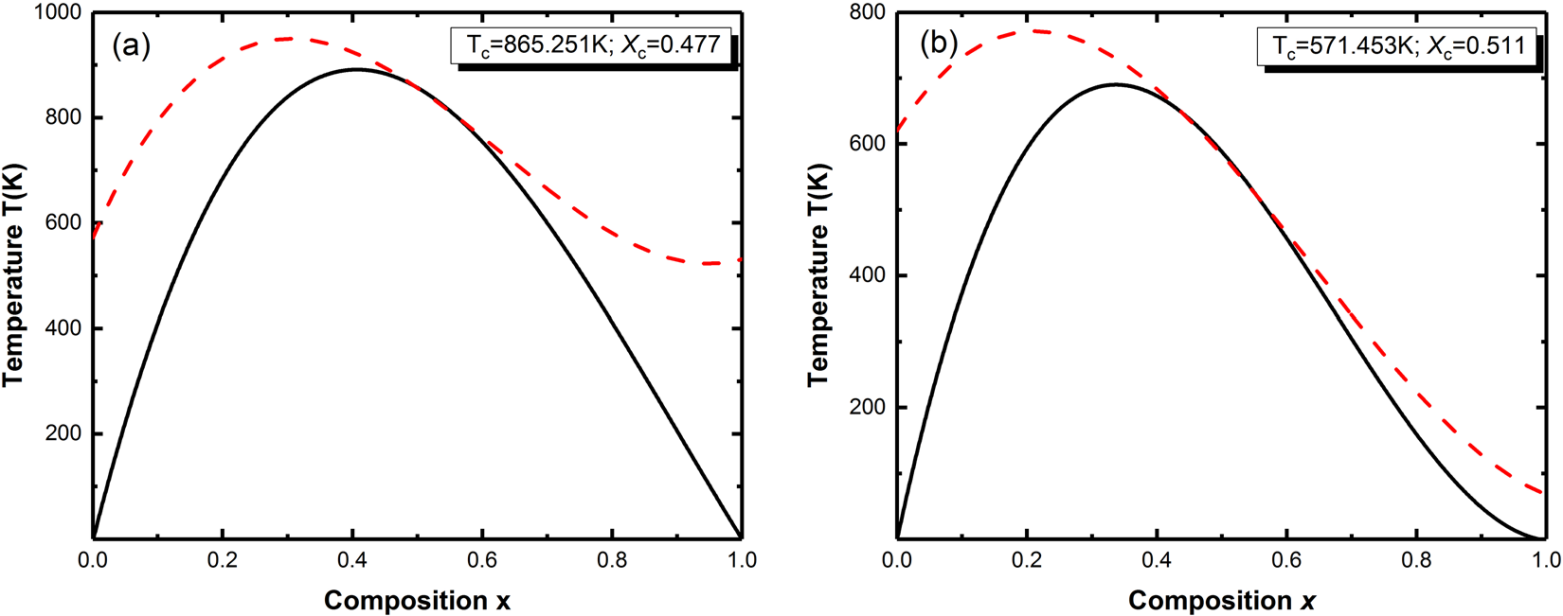
*T–x* phase diagrams of InN_1−x_Bi_x_ in (**a**) ZB and (**b**) WZ phases, respectively. The dotted line represents the binodal curve and the solid line represents the spinodal curve.

Figure 4 depicts the *T-x* phase diagrams for InNBi in ZB and WZ phases where the dotted lines represent for binodal curves and the solid lines for spinodal curves. The binodal curve is calculated by the common tangent line touched the Δ*G*_*m*_ curve, while the spinodal curve, which describes the equilibrium solubility limit, is determined from the second derivative of Δ*G*_*m*_. For ZB and WZ phases, the broad regions surrounded by the spinodalcurves show the miscibility gap and the region between the spinodal and binodal curves is of the metastable phase. The metastable phase could be formed from the thermodynamically unstable phase due to the combination between slow decomposition kinetics and rapid quenching. The miscibility gap disappears at *T*_*c*_ (the critical temperature) = 865.251 K, *x*_*c*_ = 0.477 (the critical composition) for ZB phase and at *T*_*c*_ = 571.453 K, *x*_*c*_ = 0.511 for WZ phase. The *T*_*c*_ in ZB is larger than that in WZ, while the *x*_*c*_ of ZB is smaller than that of WZ phase. Compared to the *T*_*c*_-values of other III-V alloys, such as InGaN of computed ZB *T*_*c*_ = 1400 K^39^ and WZ *T*_*c*_ = 1590 K^40^, AlGaAs, AlGaSb, GaAsSb and AlAsSb alloys with computed *T*_*c*_ of 553, 1078, 1379, and 1693 K as reported by Hassana *et al*.^41^, InAlN *T*_*c*_ of 2350 K^39^, GaInAs and InAlAs *T*_*c*_ at 1503.11 and 1277.77 K^42^, our results are much smaller, which indicate that the InNBi alloy could be stable over a wide range of intermediate compositions at a normal growth temperature. At room temperature of 300 K, InNBi with a small fraction of Bi is metastable in both ZB and WZ phases.

### Electronic properties

#### Benchmark calculations of InN bandgap

It has been shown that the theoretical accuracy of calculated III-V energy bandgap, VB and spin-orbit split-off depends on the functional or correction potential including screening effects and SOC^43, 44^. Thus, we validate our results using three sets of potentials: GGA-PBE, hybrid function of Heyd-Scuseria-Ernzerh (HSE06)^45, 46^ and MBJLDA. The calculated bandgap of InN in ZB phase is zero from GGA-PBE, 0.409 eV from HSE06 and 0.757 eV from MBJLDA which gives a value of 0.818 eV in WZ phase. In comparison with other theoretical values, 0.753 eV by PW-PP^47^ and 0.700 eV by LDA + C^48^ for ZB phase, 0.850 eV by LDA + C^48^ and 0.736 eV by LMTO^49^ for WZ phase, and the experimental value of 0.7–0.9 eV^3, 4, 50, 51^, the GGA-PBE bandgap is not reliable, the expensive HSE06 gives better but still an unsatisfied bandgap and the result by using the MBJLDA potential, which is as cheap as GGA-PBE, is in very good agreement with experimental and other theoretical results. Thus, the more efficient MBJLDA is used for further computations.

#### Electronic band structures of InNBi alloy in ZB and WZ phases

**Figure 5:**
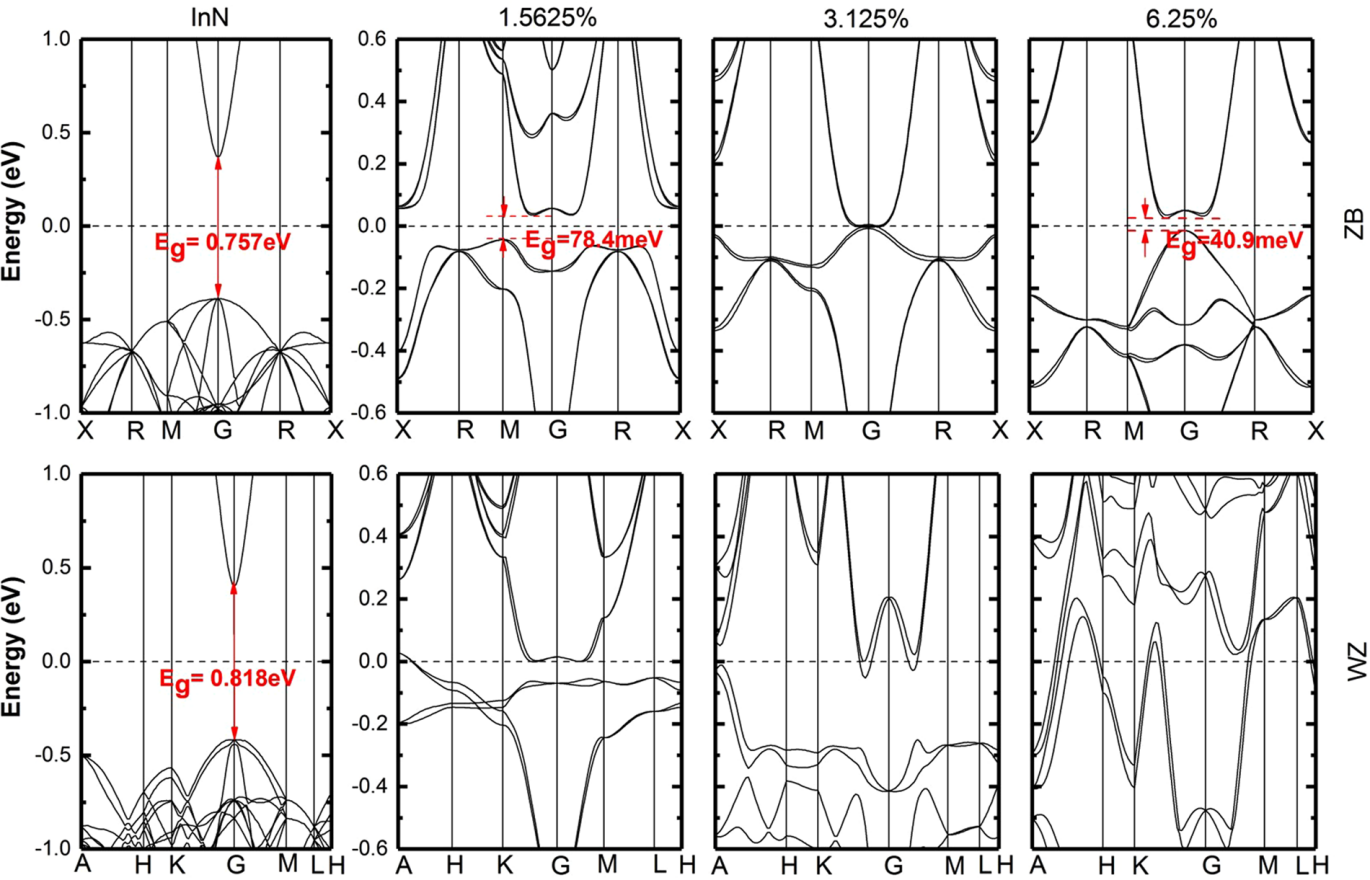
Electronic band structures of InN_1−x_Bi_x_ in ZB and WZ phases for *x* = 0, 1.5625%, 3.125% and 6.25% with MBJLDA potential.

Figure 5 depicts the electronic band structures of Bi composition at 0, 1.5625%, 3.125% and 6.25% in ZB and WZ phases. At *x* = 0.015625, the ZB-InNBi alloy has an indirect bandgap of 78.4 meV, but the WZ alloy presents semimetal characteristic with two bands crossing the Fermi level around the A-point in Brillouin-zone. The bandgap at the Γ-point is about 0.2 eV and 0.1 eV for ZB and WZ phase, respectively, giving reduction in bandgap at about 0.56 eV (356 meV/%Bi) for ZB-InNBi and 0.72 eV (460 meV/%Bi) for WZ-InNBi. This strong bandgap shrink behavior is much larger than the calculated bandgap bowing 105 meV/%Bi of InPBi^17, 52^ and 90 meV/%Bi of GaAsBi^53^. It implies much more sensitive electronic properties of this InNBi compared with other III-V materials. At *x* = 0.03125, the ZB-InNBi does not present a bandgap and the WZ-InNBi shows a semi-metallic bandgap, while at *x* = 0.0625, the ZB-InNBi band structure shows an indirect bandgap of 40.9 meV and the WZ-InNBi maintains the metallic band structure. Electronic band structures at higher *x* are also obtained but not given by graphs for the sake of concise. On the whole, as summarized in Table 3, the ZB-InNBi shows a bandgap change from direct to indirect firstly, and then an oscillation between semi-metallic and indirect bandgap for Bi composition up to 25%, and in the end a constant metalized property for higher Bi compositions. On the other hand, the WZ-InNBi shows the direct semi-metallic nature ever since 1.56% Bi is incorporated into the pristine WZ-InN. It should be noted that the semi-metallic and indirect bandgap alternation behavior of ZB-InN_1−x_Bi_x_ with *x* between 1.5625% and 25% is different from that of AlN_1−x_Bi_x_^54^ alloy, where the Bi incorporation leads to the transition from indirect (AlN) to direct bandgap (AlN_1−x_Bi_x_). This kind of bandgap variation for ZB-InNBi alloy could have much contribution for improving the optoelectronic properties of this material. The Bi atom has a larger ionic radii than that of N atom and the largest difference in electronegativity. Previous theoretical studies have demonstrated that the bigger the difference in atom size and electronegativity between the pristine III-V compound and its bismuth-containing counterpart, the more pronounced the effects^17^. Also due to the inaccuracy of the MBJLDA method (±0.1 eV), the calculated bandgap value smaller than 0.1 eV should be interpreted cautiously. The MBJLDA only gives a trend of band structure evolution with increasing Bi composition and the results shown here indicate rapid bandgap reduction in both ZB and WZ InNBi. This property is of particular interest in making use of InNBi for device applications. Adding a small amount of Bi atoms less than 2% in InN can tune transition wavelength well into far-IR or even THz regime. The arrangement of Bi atoms in ZB-InNBi alloy induces different average In-Bi bond lengths and Bi-Bi interaction also has a certain effect on the band structure, the detail discussions are given in the next section for two Bi compositions of 6.25% and 25%.

**Table 3:** Calculated bandgap (*E*_*g*_/eV), the value of Г- Г (eV) and characteristics of InNBi with increasing Bi composition in ZB and WZ phases.

*x*	ZB			WZ		
	*E*_*g*_	Г- Г	Characteristic	*E*_*g*_	Г- Г	Characteristic
0	0.757	0.757	direct band gap	0.818	0.818	direct band gap
0.015625	0.078	0.201	indirect band gap	−0.028	0.084	semi-metal
0.03125	0.000	0.000	semi-metal	−0.041	—	semi-metal
0.0625	0.041	0.064	indirect band gap	—	—	metalized
0.125	—	—	semi-metal	—	—	metalized
0.25	0.218	—	indirect band gap	—	—	metalized
0.50	—	—	metalized	—	—	metalized
0.75	—	—	metalized	—	—	metalized
1.00	—	—	metalized	—	—	metalized

#### Clustered and uniform band structures of ZB-InN_1*−*x_Bi_x_ at *x* = 6.25% and 25%

**Figure 6:**
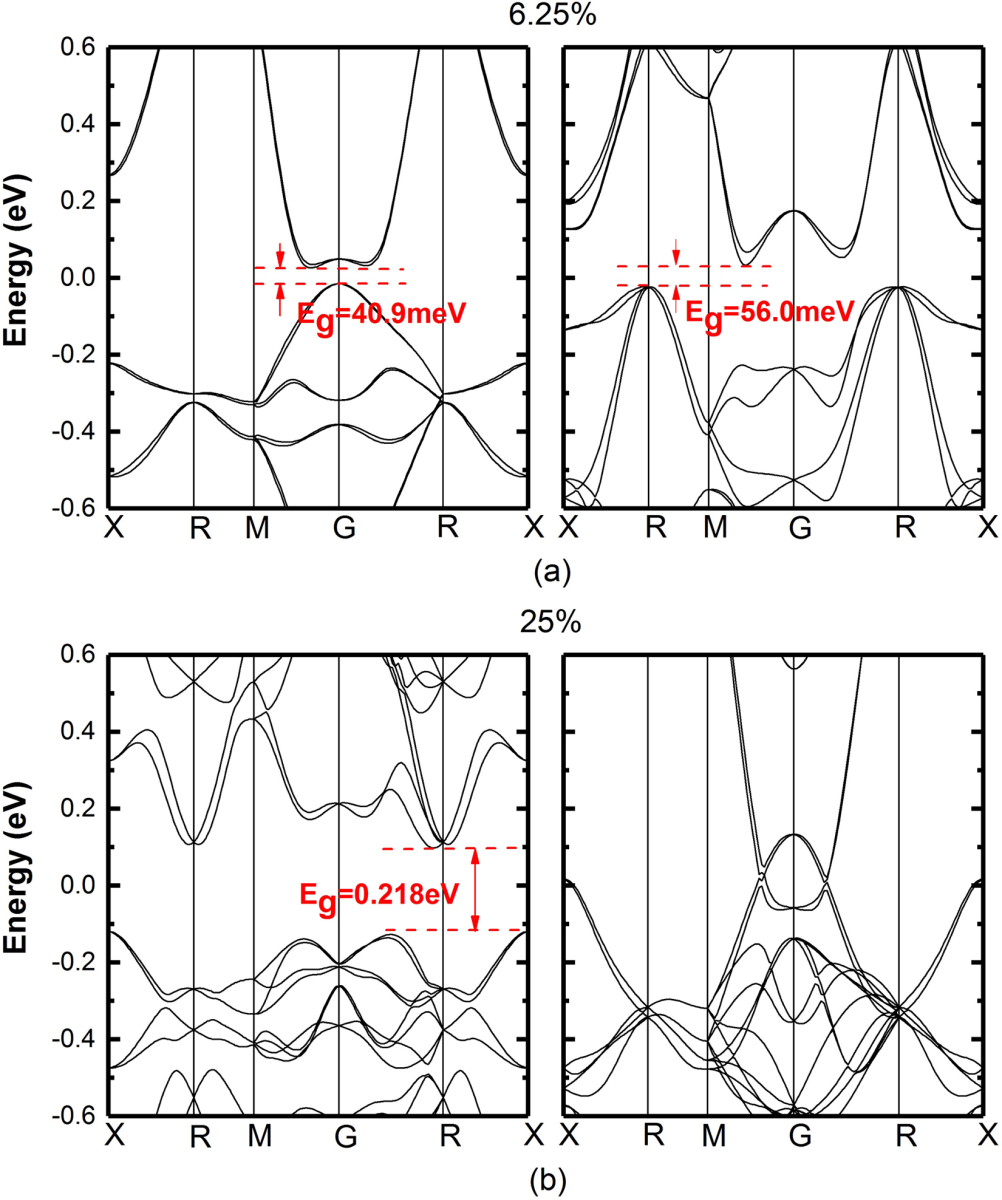
Electronic band structures for Bi clustered (left panel) and uniformly (right panel) distributed ZB-alloys of (**a**) InN_0.9375_Bi_0.0625_, and (**b**) InN_0.75_Bi_0.25_ alloys.

ZB-InNBi alloy with distributed Bi atoms in clusters presents an interesting bandgap alteration and this peculiarity is examined compared with another extreme atomic arrangement where the distribution of Bi atoms is as uniform as possible over the supercell (uniform alloy)^28^. The arrangement effect is explained by comparisons of the band structures shown in Fig. 6 and of density of states (DOSs) shown in Supplementary Fig. S2 at *x* = 6.25% and 25% Bi composition, respectively.

Figure 6(a) shows the band structures of InNBi with 6.25% N replaced by Bi in cluster form (left panel) and uniform (right panel) arrangements. The band structure of Bi clusters shows a quasi-direct energy band with a small bandgap of 40.9 meV (22.1 μm) while the uniform one reveals an indirect bandgap of 56.0 meV. The small energy of 23 meV between the minimum of CB and the Γ-point implies that electrons can be easily filled up to the Γ-point leading to efficient light emission for clustered InNBi. For InN_0.75_Bi_0.25_ with 25% Bi composition shown in Fig. 6(b), the clustered band has an indirect bandgap of 0.218 eV while an uniform one reveals metallic. This result suggests that the bandgap can be opened after Bi clusters being introduced. Previous investigation has demonstrated that the Bi substitution would lead to a broadening of these states near the Γ-point causing the bandgap reduction^55^. And another important factor, which lattice distortion and disparity in electronegativity from the large mismatch of the host and anions can not be neglected. Formation of Bi clusters breaks the crystal periodicity and introduces strong localization. The possible existence of the clustered alloys at 6.25% and 25% could result from the significant effects on the geometric configuration and electronic characteristic generated by Bi dimer and in particular octamer in bulk phases^21, 56, 57^. The volume of In surrounding the Bi atom is greatly increased by the lattice mismatch, which assists the Bi in generating a trap for hole. Compared to the uniform distribution of Bi atoms, the Bi clustering weakens the effect of structure distortion. Thus, the procedures to obtain a specific Bi composition and a specific Bi arrangement are of great importance to engineer band structures and select candidates for far-IR and THz applications. InNBi thin films and heterostructures can be epitaxially grown on InN templates. A small amount of Bi will introduce compressive strain. Depending on the Bi composition of any significance for middle-IR and THz devices, the critical thickness can be in the range of tens nm up to a few mm.

## Conclusions

The structure and thermal-stability for ZB and WZ-InNBi alloys are investigated by using GGA-PBE and electronic properties of these alloys are investigated by using MBJLDA. The calculated lattice parameters of InNBi in both phases increase with increasing Bi composition and a decrease is found for bulk modulus, indicating a non-linear variation dependence of the lattice parameters and bulk modulus on Bi composition. Thermal stability is analyzed from chemical potential based formation energy and thermodynamics properties for two phases, and the formation energy is shown almost independent of the chemical potential difference between N and Bi. The temperature-composition phase diagrams of ZB- and WZ-InNBi show broad miscibility gaps and low critical temperatures, indicating that InNBi alloy is stable over a wide range of intermediate compositions at a normal growth temperature. For electronic properties, we observe a strong shrink in bandgap of about 350–460 meV/%Bi at *x*_*Bi*_ = 1.5625% for both phases. The bandgap of WZ phase closes for Bi composition higher than 1.5625% while other peculiar electronic properties are shown for the ZB phase. The Bi centered ZB InNBi alloy presents a change from direct bandgap to indirect bandgap up to 1.5625% Bi and then an oscillation between indirect bandgap and semi-metallic for 1.5625% Bi to 25% Bi, and finally to metallic for higher Bi compositions. This effect can be explained by the mismatch of atomic size between N and Bi as well as the hybridization of *p*-orbitals. The VBs hybridized with occupied Bi *p*-orbitals contribute to the reduction of bandgap, where largely the Bi *p*_*y*_-states are hybridized with Bi *p*_*z*_-states. The Bi clustered and uniform phase with the same Bi composition can have salient differences in band structures. This work provides a new direction in this important InNBi alloy as an unconventional active material to extend transition wavelength and close the bandgap for III-nitrides. By adding Bi atoms in InN and control their incorporation form (in cluster or uniform), it is attractive to engineer electronic properties to achieve low bandgap device applications, such as future-generation solar cells, for the nitride alloys can cover the whole solar spectrum range.

## Electronic supplementary material

Supplementary information 
