# Supplementary material for: Closing the bandgap for III-V nitrides toward mid-infrared and THz applications
Source: Sci Rep. 2017 Sep 6;7:10594. doi: 10.1038/s41598-017-11093-4 (PMC5587590; doi:10.1038/s41598-017-11093-4)
Supplement: Supplementary file 1 — Supplementary information [file 41598_2017_11093_MOESM1_ESM.docx]

**Supplemental Information for “Closing the bandgap for III-V nitrides toward mid-infrared and THz applications”**

Pengfei Lu, Dan Liang, Yingjie Chen, Chunfang Zhang, Ruge Quhe, Shumin Wang

E-mail: [photon.bupt@gmail.com](mailto:photon.bupt@gmail.com), shumin@chalmers.se,

**I. Variation of lattice constants compared to Vegard’s law**

The plots in Fig.S1 display the lattice constant *a* and *c* as a function of Bi composition together with Vegard’s law [1], where a clear deviation from this law is shown. Upon rising Bi composition there is an increase trend for the lattice constant with the total increment of 1.749 Å for *a* in ZB and total increment of 1.329 and 2.044 Å for *a* and *c* in WZ, respectively. The obtained bowing coefficient of the lattice constant *a* and *c* represented by *δa* and *δc*, defined as the largest deviation from Vegard’s law, are 1.150 Å for ZB, 1.523 Å in *a* and -1.516 Å in *c* for WZ, respectively. Note that the lattice constant of ZB phase has a smaller deviation from Vegard’s law than those of WZ phase, mainly due to relaxation of the In-N and In-Bi bond lengths in InNBi alloys. Several previous experimental [2] and theoretical [3] works have demonstrated that Vegard’s law is no longer valid for the systems with strong lattice mismatch as III-N nitride alloys, due to a large difference in anion’s atomic radius which is also confirmed by this work.


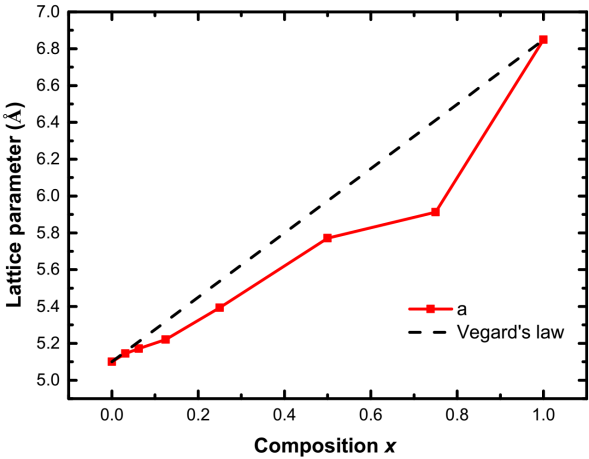

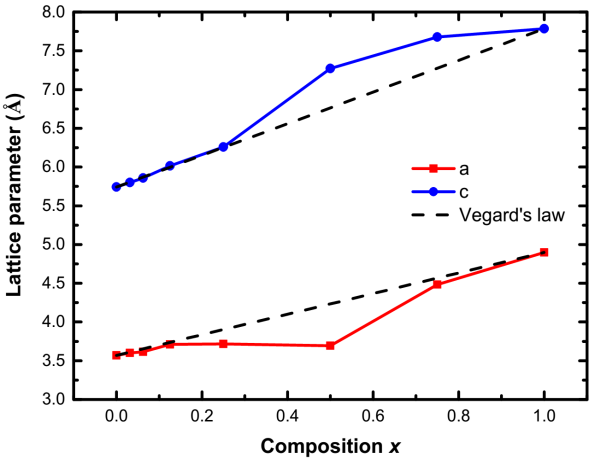


1. (b)

**Figure. S1** Variation of lattice constants *a* and *c* as a function of Bi composition for (a) ZB and (b) WZ phases, respectively.

**II. The density of states at *x*=6.25% and 25% Bi composition**

The total (TDOS) and partial (PDOS) density of states for *x*=0, 0.0625 and 0.25 in clustered and uniform Bi arrangements are shown in Fig. S2, where the In-*s*/*p* and Bi-*s*/*p* states are given and the In-*d* and Bi-*d/f* states are neglected due to the weak influence of the *d*-orbitals. The TDOS of 6.25%-Bi clustered and uniform InNBi alloy, as shown in the left panel of Fig. S2(a), are quite different from that of the binary in the vicinity of Fermi level, which indicates the effect from Bi incorporation. Clearly seen from the right panel of Fig. S2(a), the clustered valence band maximum (VBM) and conduct band minimum (CBM) are largely dominated by Bi-*p_y_* or Bi-*p_y_* hybridized with Bi-*p_z_* states together with certain contributions from the *p*-orbitals of In and N, while the uniform ones are mainly from Bi-*p_y_* together with certain contributions from the *p*-orbitals of In. Besides, the clustered VBM exhibits a shoulder structure while the uniform one has a single peak, and the CBMs of both distributions are similar due to the same contributions from *p*-orbitals of Bi, In and N. As indicated in Fig. S2(a), the bandgaps of the two arrangements are comparatively small with some indeterminacy and consequently there are no significant energy gaps indicated in TDOS although the uniform one has a lower density compared to the clustered one at the Fermi level. The similar illustration analysis for InN_0.75_Bi_0.25_ is presented in Fig. S2(b), where the clustered InN_0.75_Bi_0.25_ exhibits a small energy gap and relatively smooth but small TDOS and PDOS, and the uniform InN_0.75_Bi_0.25_ shows several sharp peaks in VB probably due to a high degree of electron localization. Numerous electronic structure calculations and experiments have confirmed the hybridization mechanism in Bi-containing alloys [4-8], i.e. the reduction of bandgap is caused by the VB hybridization with occupied Bi *p*-orbitals and that the compositional disorder plays a major role at a high Bi composition. The hybridization of VB with Bi orbitals can be inferred from the transport measurements which show a reduction of hole mobility by an order of magnitude compared to the host materials while the electron mobility is much less affected [9].


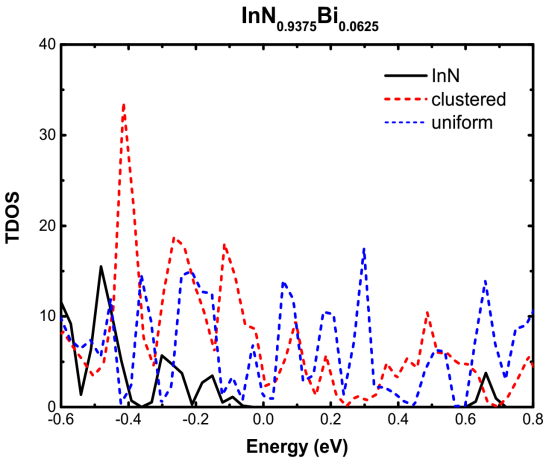

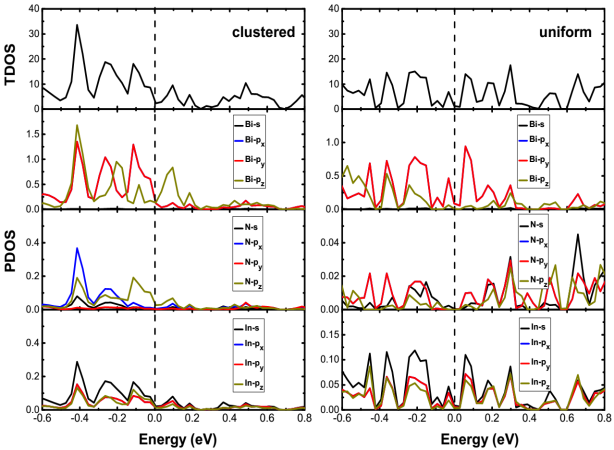


(a)


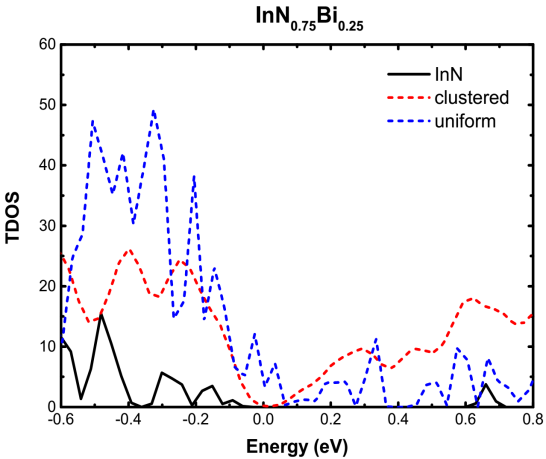

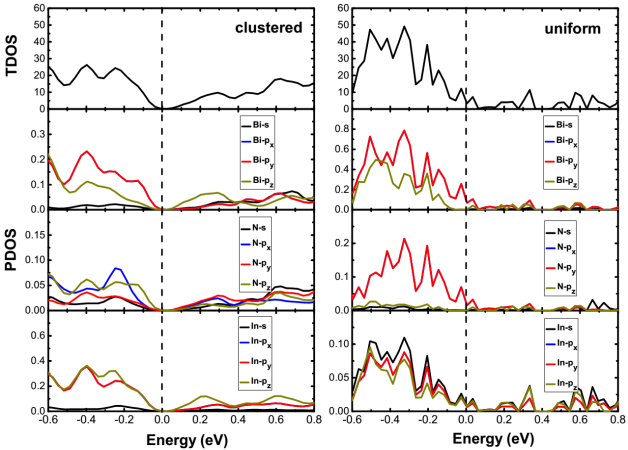


(b)

**Figure. S2** Total and partial density of states for clustered and uniform arrangements in (a) 6.25%-Bi InNBi alloy and (b) 25%-Bi InNBi alloys, together with TDOS of pristine InN for comparison.

**References**

1. Vegard, L. Formation of mixed crystals by solid-phase contact. *Z. Phys.* **17**, 2 (1921).
2. Fan, W. J. Determination of nitrogen composition in GaN_x_As_1−x_ epilayer on GaAs. *J. Cryst. Growth*. **268**, 470 (2004).
3. Carrier, P., Wei, S. H., Zhang, S. B. & Kurtz, S. Evolution of structural properties and formation of N-N split interstitials in GaAs_1−x_N_x_ alloys. *Phys. Rev. B*. **71**, 165212 (2005).
4. Kini, R. N. *et al*. Effect of Bi alloying on the hole transport in the dilute bismide alloy GaAs_1−x_Bi_x_. *Phys. Rev. B*. **83**, 075307 (2011).
5. Nargelas, S., Jarasiunas, K., Bertulis, K. & Pacebutas, V. Hole diffusivity in GaAsBi alloys measured by a picosecond transient grating technique. *Appl. Phys. Lett*. **98**, 082115 (2011).
6. Beaton, D.A., Lewis, R.B., Masnadi-Shirazi, M. & T. Tiedje. Temperature dependence of hole mobility in GaAs_1−x_Bi_x_ alloys. *J. Appl. Phys.* **108**, 083708 (2010).
7. Cooke, D.G., Hegmann, F. A., Young, E. C. & Tiedje, T. Electron mobility in dilute GaAs bismide and nitride alloys measured by time-resolved terahertz spectroscopy. *Appl. Phys. Lett*. **89**, 122103 (2006).
8. Usman, M., Broderick, C. A., Lindsay, A. & O’Reilly, E. P. Tight-binding analysis of the electronic structure of dilute bismide alloys of GaP and GaAs. *Phys. Rev. B.* **84**, 245202 (2011).
9. Bannow, L.C. *et al*. Configuration dependence of band-gap narrowing and localization in dilute GaAs_1−x_Bi_x_ alloys. *Phys. Rev. B.* **93**, 205202 (2016).
